# Cutaneous Myiasis: An Unusual Cause of Posterior Auricular and Occipital Lymphadenopathy in a Toddler

**DOI:** 10.7759/cureus.7581

**Published:** 2020-04-07

**Authors:** Carole Mensah, Andrew P Tavares, Brigitte Jahoor, Cynthia Echefu-Nnaji, Adebayo Adeyinka

**Affiliations:** 1 Pediatrics, The Brooklyn Hospital Center, Brooklyn, USA

**Keywords:** myiasis, bot fly, pediatric

## Abstract

Cutaneous myiasis is a condition that involves an infestation of fly larvae into human tissue, most commonly caused by Dermatobia (D.) hominis or the bot fly. While this is a condition most commonly seen in tropical regions of the globe due to increased travel to endemic regions, physicians must increasingly be aware of this as a potential diagnosis. In addition, there is minimal literature on cutaneous myiasis in the pediatric patient and its potential associated symptoms. This case report thus highlights a toddler that presented to our facility with a raised, erythematous scalp lesion and associated preauricular and occipital lymphadenopathy. Of note, the patient had a recent travel history to Belize, an endemic area where she was likely infected. As there are multiple other differentials for not only scalp swellings in the pediatric population, in addition to regional adenopathy, a high index of suspicion was needed to make the diagnosis. Ultrasound was the imaging modality used to visualize the fly larva, and surgical excision was the mechanism of treatment. Thus, this case highlights a unique presentation of cutaneous myiasis in a toddler and aims to add to the growing body of literature on a condition likely to be encountered by physicians at a greater frequency.

## Introduction

Cutaneous myiasis is described as the infestation of human tissue by dipterous fly larvae [1-2]. Dermatobia (D.) hominis is the most common bot fly species that affects humans [3]. The human bot fly is not endemic to the United States, as their habitat is primarily localized to tropic and subtropics regions, including Central and South America, Africa, and the Caribbean [4]. However, due to increased travel to and from endemic areas, physicians must increasingly be aware of this as a potential infectious process in their patients. The mechanism by which the bot fly infects a human source pivots on an understanding of their life cycle. The adult bot fly captures a blood-sucking arthropod (e.g., mosquito or tick) and lays its eggs on the vector that will, in turn, take a blood meal from the human host. The larvae developing in the eggs will enter the human subcutaneous tissue where they may feed for four to 10 weeks [2-3]. When fully mature, the larvae exit the skin through an open pore, fall to the soil, and pupate [2]. In the human host, it is during the maturation period in which the patient will often develop a raised, erythematous lesion on their skin that gradually enlarges as the larvae grow [2-3]. The lesions are often intermittently painful and typically have an open-air pore. It is important to note, however, that the signs and symptoms of cutaneous myiasis will differ depending on the maturation stage and location of the infestation. While, in recent years, there has been an increasing number of reports of myiasis seen by physicians in North America, few cases have been documented in the pediatric population. Thus, we present to you a unique case of myiasis in a pediatric patient.

## Case presentation

A three-year-old female with a history of recent travel history to Belize and Guatemala presented with swelling and pruritus of the occipital scalp two weeks after returning to the United States. She was seen in the emergency room of a local hospital. During her trip, the patient, accompanied by her family, visited many forests, lakes, rivers, and sulfur hot springs. While in those countries, her mother reported multiple mosquito bites despite taking protective measures. The patient’s grandmother had similar localized swelling and pain on the lower extremity, which was incised and drained at the emergency department at an outside institution. The emergency department physician reported extracting an unknown larva from her grandmother’s wound and recommended that the patient be evaluated due to similar symptoms. At the emergency department of an outside facility, physical examination was remarkable for a 1x1 cm occipital scalp swelling associated with associated pruritus. Unsuccessful incision and drainage were performed and the patient was then transferred to our pediatric inpatient unit for further management.

**Figure 1:**
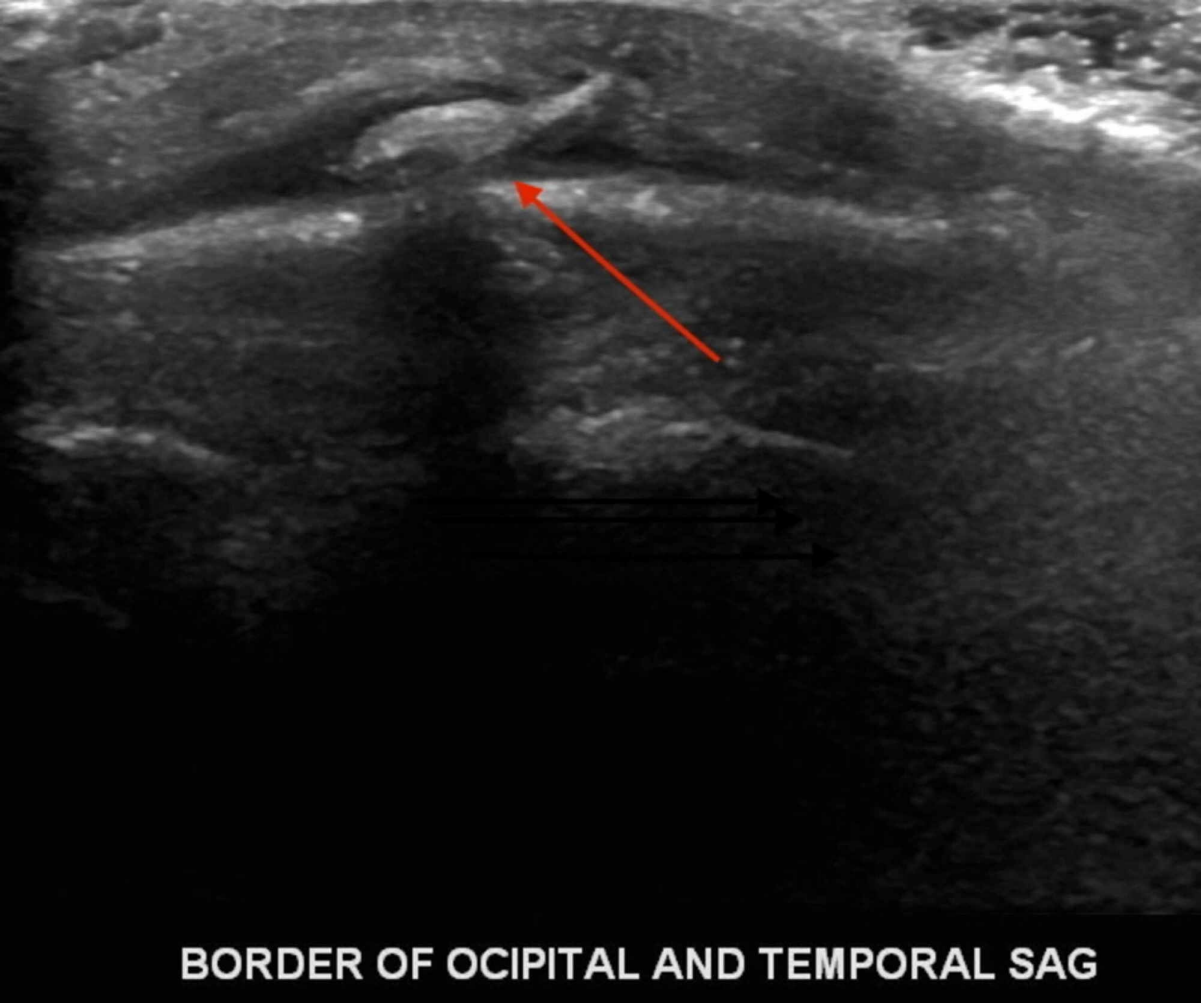
Ultrasound of scalp showing foreign body

**Figure 2:**
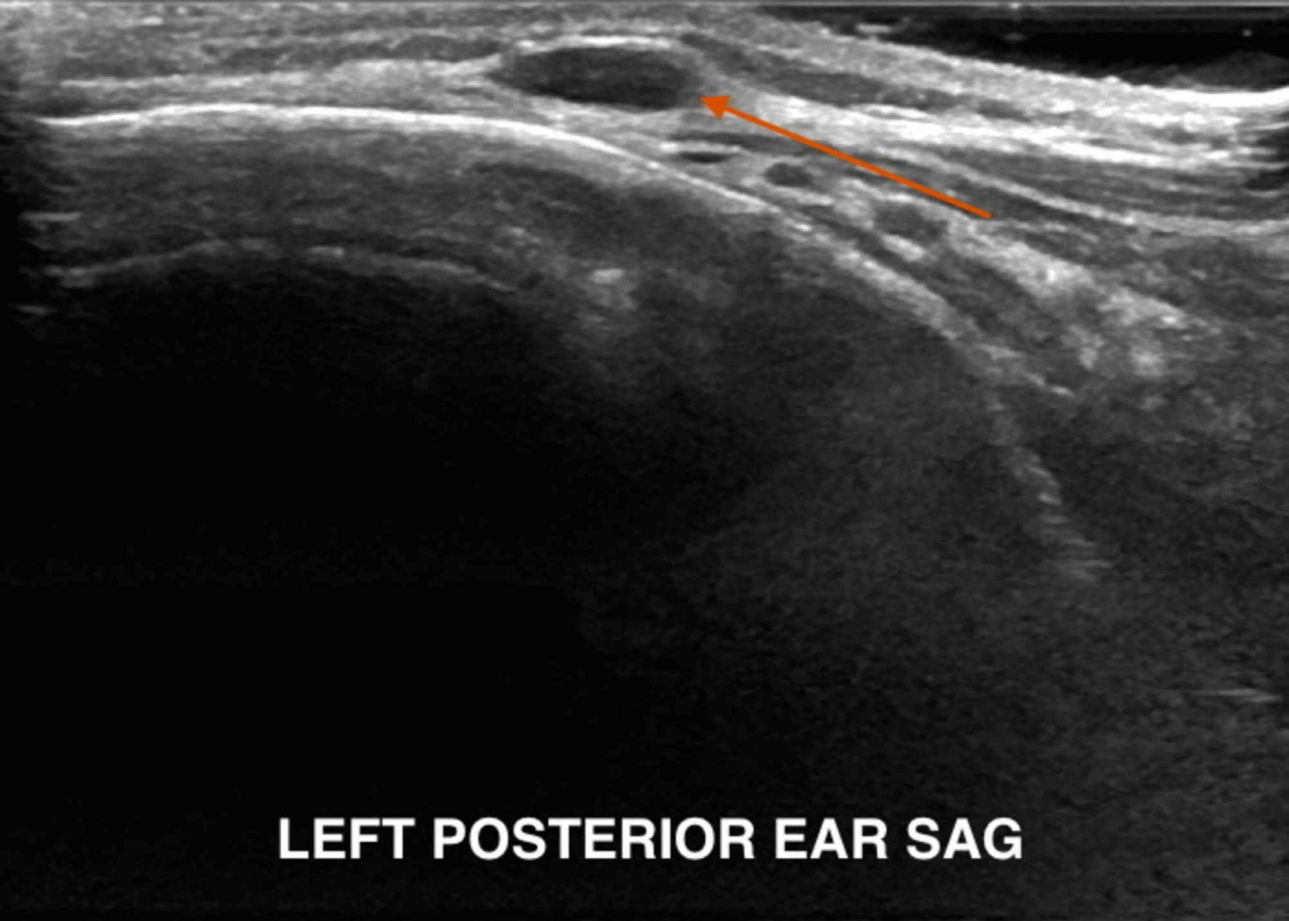
Posterior auricular adenopathy

**Figure 3:**
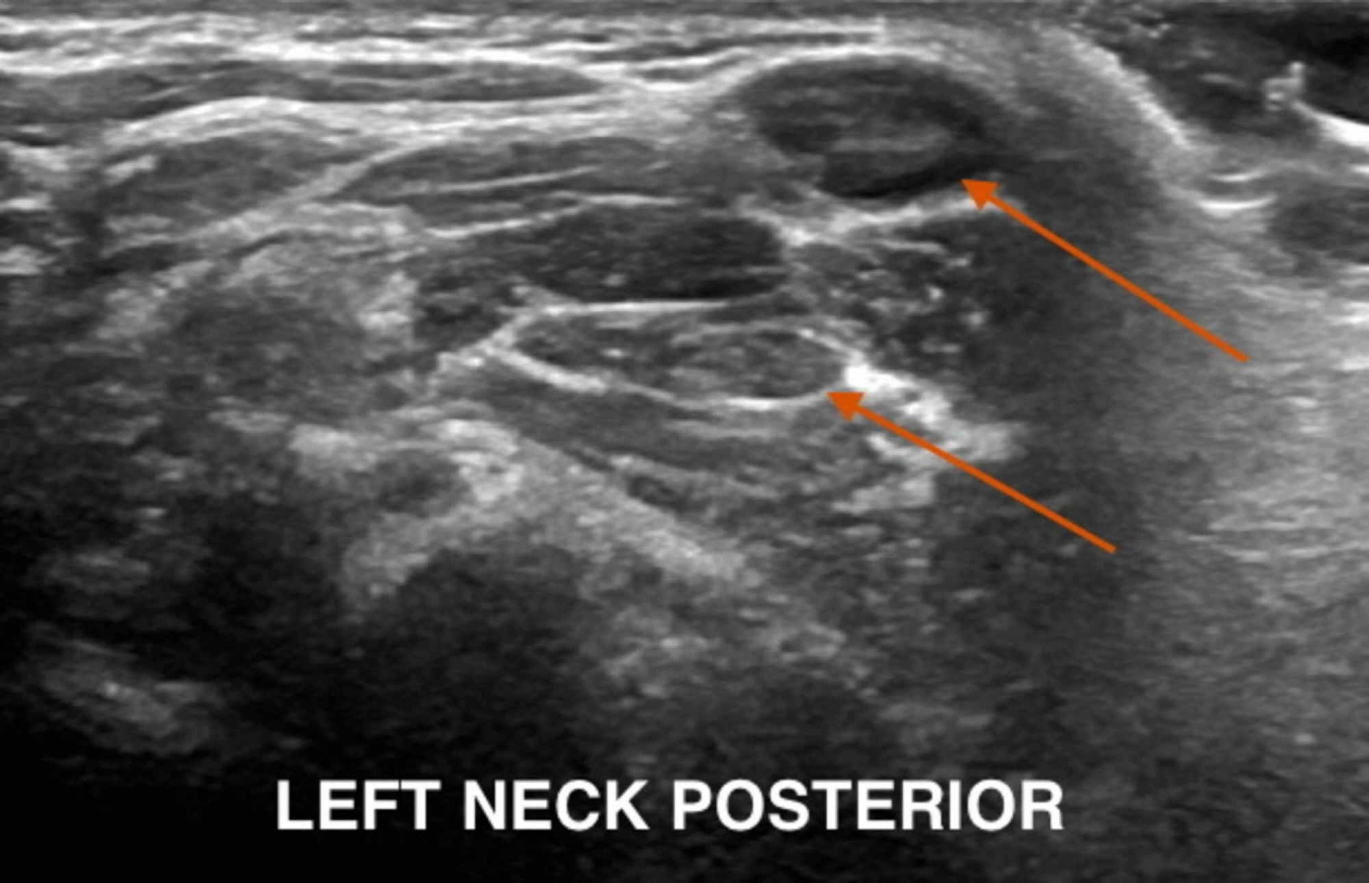
Posterior cervical adenopathy

On presentation, the physical examination was significant for a 1 cm x 1 cm open laceration on the posterior scalp with surrounding swelling and erythema; no fluctuance or drainage of the wound was appreciated. The patient also presented with left-sided postauricular lymphadenopathy and bilateral occipital lymphadenopathy with the largest measuring 1x1 cm, firm, non-tender to palpation. Ultrasound was done to rule out other causes of scalp swelling (abscess, cellulitis, simple cyst) and to confirm the presence of a foreign body. Ultrasound results showed an echogenic structure in the subcutaneous tissue measuring 8x5x3 mm, which may represent the foreign body (Figure 1) with a posterior left neck lymph node measuring up to 0.9x0.8x0.5 cm and a left peri-auricular lymph node measuring 0.8x0.7x0.3 cm (Figures 2-3).

A layer of thick petroleum jelly was applied to the site, occluding the pore to deprive the larva of air. Surgery was then consulted for incision and drainage of the swelling. Under local anesthesia, a 3 cm x 1 cm incision was made on the skin of the posterior scalp; exploration of the wound was done to identify and remove the foreign body (Figure 3).

**Figure 4:**
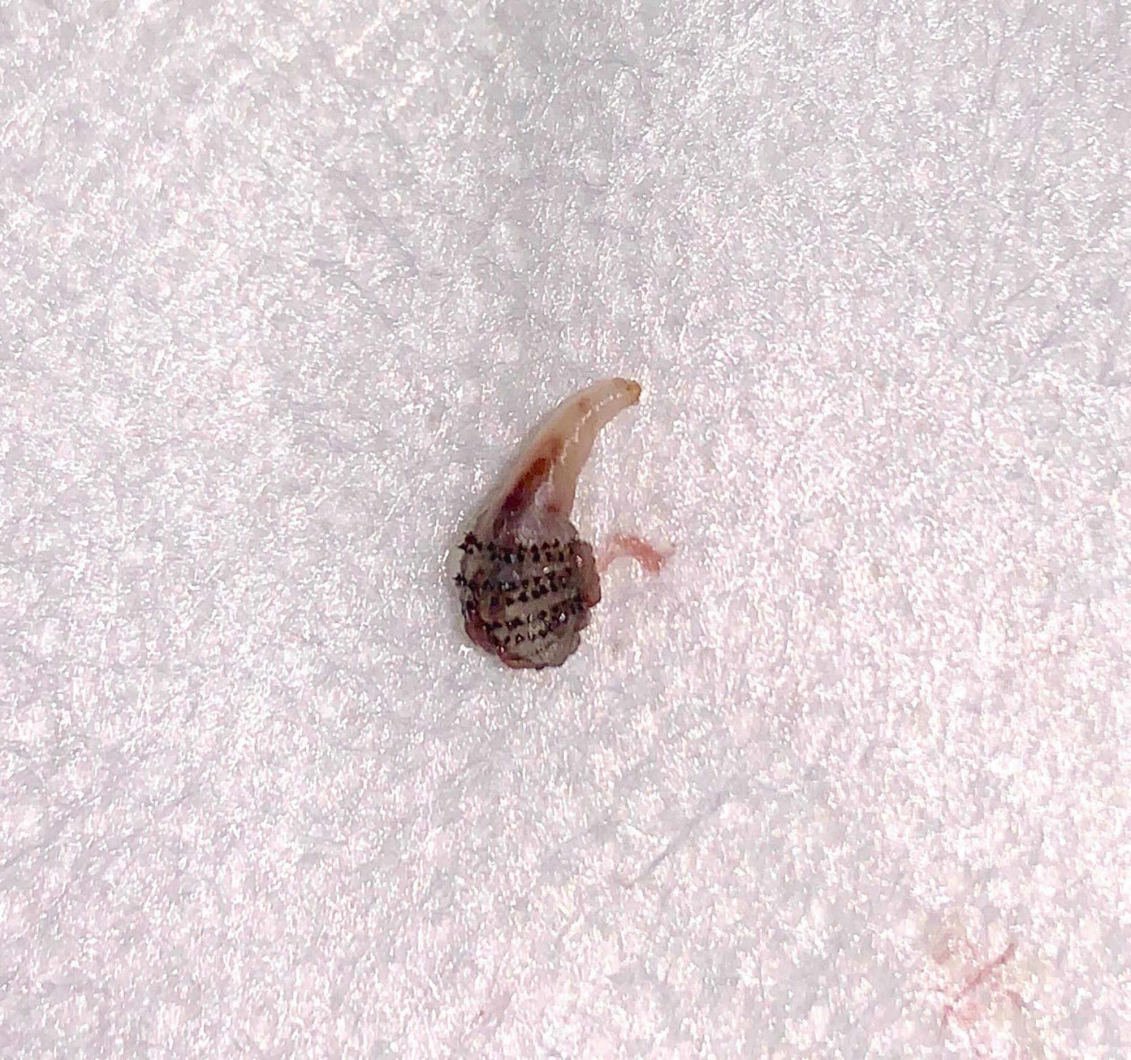
Excised bot fly larva

The foreign body was sent to pathology for identification. Pathology results showed fly larva (myiasis causing), which confirmed the diagnosis (Figure 4).

## Discussion

Among pediatric patients, case reports of myiasis caused by the human bot fly (D. hominis) are very rare; an extensive literature review revealed only around 10 cases reported worldwide. As in our patient, the most common site of bot fly myiasis is the scalp, a region not typically covered and amenable to the inciting insect bite required for inoculation [5-10].

Exposure to D. hominis is almost exclusively found in those with a history of travel to South or Central America [8,11]. No documented cases of D. hominis myiasis acquired in the United States exist in the literature. However, a recent case report from Canada documents infection in a patient with no prior history of travel [12]. Cases such as this demonstrate that locally acquired infection in patients with no prior travel history is possible. Furthermore, recent changes in global weather patterns may lead to myiasis becoming a more common phenomenon in northern latitudes in the years to come.

Patients with cutaneous myiasis typically present due to a raised papule with a central, necrotic opening and surrounding erythema [4,8,11]. Pain and intense pruritis are common presenting symptoms [8,13-14]. Lesions are commonly accompanied by serous or serosanguinous discharge, which may be enhanced by applying pressure to the site, but purulent discharge is uncommon and points to secondary infection or an alternate diagnosis [8-9,11,13]. The lesions are most often mistaken for folliculitis, dermal cysts, cutaneous leishmaniasis, foreign objects embedded in the skin, or furuncles (hence the origin of the name “furuncular myiasis”) [8,12,14]. Due to misdiagnosis, most patients also report ineffective treatment with antibiotics in the outpatient setting [13-15].

On examination, a mass underlying the lesion may be felt and many patients report the sensation of something moving beneath the skin [13-15]. Besides the noticeable lesion, most patients deny any systemic symptoms, although regional lymphadenopathy has occasionally been reported in the literature [7,9].

As it pertains to our patient, the presentation was unique in that the lesion was located on the occipital scalp, with accompanying multiple post-auricular and occipital lymphadenopathy. To our knowledge, this specific distribution of lymphadenopathy secondary to bot fly infection has not previously been reported in the literature. Postauricular lymphadenopathy is most commonly noted in bacterial or fungal infections of the parieto-temporal scalp, in addition to infections caused by rubella and roseola. Occipital lymphadenopathy in the pediatric population more commonly presents with localized infections such as tenia capitis, head lice, or impetigo [16]. While the aforementioned are the most common etiologies, we suggest that cutaneous myiasis be included in the differential of pediatric patients presenting with post-auricular or occipital lymphadenopathy.

Once suspicion of bot fly infection exists, imaging modalities may be used to identify the presence of the larva beneath the skin. While computed tomography (CT) and magnetic resonance imaging (MRI) have been used in identification, recent studies indicate that ultrasonography is a superior imaging modality [8-10]. Ultrasound allows for the visualization of the larvae at an early stage and even when the typical “punched-out” lesion is not present [7]. This is especially pertinent in our patient, as an incision had been made at another institution prior to presentation, distorting the appearance of the lesion. In general, ultrasound is effective in differentiating between abscess and other causes of scalp lesions, including myiasis, and allows for the visualization of larval movement in real time [15,17]. It has even been suggested that the larval stage may be identified during ultrasonography by those with knowledge of the bot fly lifecycle [6].

Once identified, the treatment of myiasis consists of extracting the botfly larvae. As most physicians in the United States are unfamiliar with myiasis, surgical extraction, as was done in our patient, is the most commonly used means of resolving bot fly infections reported in the literature [14]. Simple extraction through the central pore should not be attempted, as the bot fly larva uses a row of spines to anchor itself in place [12]. If surgical extraction is chosen, a local anesthetic (typically lidocaine) is applied, which often paralyzes the larva, and a small incision is made, after which the larvae may be removed using forceps [7,14-15].

Following the customs of residents of botfly endemic regions, botfly larvae may alternatively be removed without the need for invasive surgical intervention [7]. Simple removal of the larva can be achieved by covering the central pore with petroleum jelly, beeswax, nail polish, raw meat, or chewing gum, thereby depriving the larva of oxygen [11,13]. After a few hours, the botfly larva will protrude through the wound site in search of air, at which point, it may be extracted using forceps. Gentle pressure may also be applied around the wound site to aid in expelling the larva [11]. If the larva suffocates prior to extruding through the wound, surgical intervention may then be required to extract it in order to prevent secondary infection [5,14]. In our patient, although petroleum was applied to the site, we suggest that not enough time elapsed to allow for the larva to expose itself sufficiently to allow for extraction.

After surgical extraction, the wound should always be thoroughly cleaned, debrided of any necrotic tissue, and closed. The role of treatment with antibiotics after extraction is debated but does not appear to be routinely necessary [11]. Rarely, secondary infections arise, most notably if only partial extraction of the larva is achieved [4,11,14]. If infection occurs, ivermectin is the treatment of choice. Of interest, a case report identified the extraction of a D. hominis larva without the need for surgical excision after a single dose of ivermectin and is an interesting avenue for future research [18]. After removal, lesions typically resolve within a matter of days to weeks, with no sequela [8].

The diagnosis of cutaneous myiasis is important to consider in pediatric patients presenting with cutaneous lesions similar in appearance to an abscess, with a history of recent travel to bot fly endemic areas. Generally, clinical suspicion for myiasis in pediatric patients in the United States is low because: (i) The botfly is not an endemic species, and without a specific history of travel to an endemic area (i.e., Central America), probability of exposure is almost non-existent, (ii) Presentation varies significantly in the size and location of lesions and associated symptoms, and (iii) Based on the literature review, the condition in children appears to be much rarer than in adults.

## Conclusions

Cutaneous myiasis secondary to bot fly infection is a rare clinical entity in the United States and, as such, is not typically included in the list of differentials for superficial cutaneous lesions in pediatric patients. Ultrasonography is recommended in identifying the presence of bot fly larvae, regardless of their size or stage of development. Although this condition can be easily treated via extraction of the larva through surgical means, increased knowledge of the condition demonstrates that non-surgical extraction is technically simple and prevents unnecessary invasive procedures, with a definitive cure. Although it may represent a rare clinical entity in any patient presenting with a cutaneous lesion of the scalp, with associated regional lymphadenopathy, myiasis should be included in the differential diagnosis. Overall, thorough travel history and a high index of clinical suspicion is necessary to prevent an unnecessary delay in diagnosis, with the continuation of lancinating pain and irritation, and undue frustration on behalf of the family.
